# Structural Insights into the Neutralization Properties of the Fully Human, Anti-interferon Monoclonal Antibody Sifalimumab*

**DOI:** 10.1074/jbc.M115.652156

**Published:** 2015-04-29

**Authors:** Vaheh Oganesyan, Li Peng, Robert M. Woods, Herren Wu, William F. Dall'Acqua

**Affiliations:** From the Department of Antibody Discovery and Protein Engineering, MedImmune LLC, Gaithersburg, Maryland 20878

**Keywords:** autoimmune disease, epitope mapping, interferon, monoclonal antibody, nuclear magnetic resonance (NMR), protein expression, sifalimumab, structural biology, systemic lupus erythematosus, x-ray crystallography

## Abstract

We report the three-dimensional structure of human interferon α-2A (IFN-α2A) bound to the Fab fragment of a therapeutic monoclonal antibody (sifalimumab; IgG1/κ). The structure of the corresponding complex was solved at a resolution of 3.0 Å using molecular replacement and constitutes the first reported structure of a human type I IFN bound to a therapeutic antibody. This study revealed the major contribution made by the first complementarity-determining region in each of sifalimumab light and heavy chains. These data also provided the molecular basis for sifalimumab mechanism of action. We propose that its interferon-neutralizing properties are the result of direct competition for IFN-α2A binding to the IFN receptor subunit 1 (IFNAR1) and do not involve inhibiting IFN-α2A binding to the IFN receptor subunit 2 (IFNAR2).

## Teaser

**Background:** We investigated the molecular basis of human IFN-α2A recognition by sifalimumab.

**Results:** We determined the structure of the complex between the Fab of sifalimumab and IFN-α2A.

**Conclusion:** The interferon-neutralizing properties of sifalimumab result from direct competition for IFN-α2A binding to IFN receptor subunit 1 and not IFN receptor subunit 2.

**Significance:** These data provide the basis for the mechanism of action of sifalimumab.

## Introduction

Interferons (IFNs) belong to the “4-helical cytokines” superfamily (1) and can be grouped into types I, II, and III. IFN-γ and IFN-λ are the only known members of the type II and III IFNs, respectively (2, 3), whereas type I IFNs constitute a family of cytokines expressed from more than 15 genes. Most notably, these include IFN-α, IFN-β, IFN-τ, IFN-κ, IFN-ϵ, IFN-δ and IFN-ω. The critical role of IFNs in modulating the host mammalian responses to infections has been well documented (4–6). More recently, IFNs have also been shown to be key immunoregulatory cytokines. As such, they play a central role in the onset of various autoimmune diseases (7, 8). Direct evidence includes the observation that autoimmune-predisposed mice deficient in the IFN-α/-β receptor exhibit significantly reduced disease manifestations such as the presence of anti-erythrocyte autoantibodies, hemolytic anemia, anti-DNA autoantibodies, and kidney disease (9). In particular, systemic lupus erythematosus, type I diabetes, and Sjögren syndrome, as well as thyroid diseases, have now been linked to the action of IFN-α (10). The existence of at least 13 subtypes within the IFN-α family (11) further complicates a thorough understanding of these pathways. To contribute to the treatment of autoimmune diseases, AstraZeneca/MedImmune has developed sifalimumab, a fully human monoclonal antibody that binds to, and inhibits the actions of multiple IFN-α subtypes.

We sought to understand the molecular basis of human IFN-α2A recognition by sifalimumab. For this purpose, we solved the x-ray crystal structure of the complex between the Fab fragment of this antibody and IFN-α2A. The structures of several type I human IFNs (*e.g.* IFN-α2A, IFN-α2B, and IFN-β), unbound or bound to a single chain Fv (scFv), have already been determined using either x-ray crystallography or nuclear magnetic resonance (NMR) (12–16). However, this study describes the first three-dimensional structure of a human type I IFN bound to a therapeutic antibody currently in human. Our data permitted us to describe in detail the corresponding interface and provide a molecular understanding the interferon-neutralizing properties of sifalimumab.

## Experimental Procedures

### 

#### 

##### Reagents, Conventions, and Illustrations

All chemicals employed were of analytical grade. The histidine-tagged recombinant extracellular domain of the IFN-α receptor 1 (IFNAR1-His_6_) was a generous gift from Sandrina Phipps (MedImmune). All antibody and antigen amino acid positions mentioned in the text were identified according to a consecutive numbering scheme. In these conditions, the Kabat-defined complementarity determining regions (CDR)^3^ (17) of sifalimumab were identified as follows: 31–35, 50–66, and 98–105 for the heavy chain (CDRH1, H2 and H3, respectively), and 24–35, 51–57, and 90–98 for the light chain (CDRL1, L2 and L3, respectively). All illustrations were prepared using PyMOL (DeLano Scientific, Palo Alto, CA).

##### Protein Expression, Purification, Crystallization, and X-ray Data Collection

Detailed purification, crystallization, and data collection procedures have been previously described (18). In short, crystals of the sifalimumab Fab·IFN-α2A complex diffracting to 3.0 Å were obtained using vapor diffusion. The orthorhombic crystals belonged to the I222 space group with unit cell parameters *a* = 134.82, *b* = 153.26, *c* = 163.49 Å. The crystals exhibited a relatively loose packing with a solvent content and Matthew's coefficient of 59.3% and 3.02 Å^3^ Da^−1^, respectively. Two sifalimumab Fab·IFN-α2A complexes were in the asymmetric part of the unit cell.

##### Structure Determination and Refinement

Diffraction images were integrated and scaled using HKL 2000 (19). Molecular replacement, refinement, and electron density calculation were completed via the CCP4 (Collaborative Computational Project Number 4) program suite (20). The crystal structure of the sifalimumab Fab·IFN-α2A complex was solved using molecular replacement and refined at 3.0-Å resolution. The search model for sifalimumab Fab consisted of the Fab portion of another antibody from AstraZeneca/MedImmune whose structure was determined at 2.17-Å resolution (21). The sequence identities between the Fab portions of sifalimumab and the search model were 95.3 and 78.6% for the light and heavy chains, respectively. The non-identical amino acids were first modeled as alanine during the molecular replacement procedure and initial refinement/model building rounds. A very clear solution was obtained for the 2 sifalimumab Fab molecules in the asymmetric unit using both PHASER (22) and MolRep (23). For the IFN-α2A portion, 3 human type I IFN structures were available in the Protein Data Bank (PDB) (24) at the time of the study (2008). These corresponded to PDB codes 1ITF (human IFN-α2A exhibiting 100% sequence identity with IFN-α2A of this study; NMR-solved), 1RH2 (human IFN-α2B exhibiting 99% sequence identity with IFN-α2A of this study, x-ray-solved at 2.9 Å resolution), and 1AU1 (human IFN-β exhibiting 39% sequence identity with IFN-α2A of this study, x-ray solved at 2.2-Å resolution). None of these 3 potential models yielded a clear molecular replacement solution with PHASER or MolRep. However, the phases obtained through the solution of both sifalimumab Fab molecules yielded very clear electron density for the proximal region of IFN-α2A. Two rounds of Fab-only refinement and model adjustment using the “O” software (25) further improved the electron density quality of the antigen and made it possible to build 3 of 5 helices manually. The resulting partial model was then superimposed on the structure of human IFN-α2B (PDB ID 1RH2), which differed from IFN-α2A by only one amino acid (R23K). The tight non-crystallographic symmetry restraints were used throughout the refinement of the model with Refmac5 (26). The substituted alanine residues were changed to their respective *bona fide* counterparts when permitted by the corresponding electron densities. The first 14 amino acids of both IFN-α2A molecules in the asymmetric unit were built last, because of the larger conformational differences. The latter may provide a reasonable explanation for not obtaining a clear solution during the molecular replacement procedure. Upon completion, the model was analyzed using the TLS Motion Determination (TLSMD) program running on its Web server (27, 28). Further refinement was carried out in TLS and restrained refinement mode using Refmac5. For this purpose, each of 3 different polypeptides were divided into 4 parts in accordance with results from the TLSMD server. More precisely, sifalimumab light chain was divided into sections corresponding to residues 1–40, 41–106, 107–177, and 178–215. Likewise, the sifalimumab heavy chain was divided into sections corresponding to residues 1–62, 63–129, 130–185, and 186–219. Finally, IFN-α2A was divided into sections corresponding to residues 1–20, 21–51, 52–113, and 114–157. The same portions were used for NCS restraints application. As we previously described (18), the IFN-α2A antigen used in this study included one extra threonine and one extra serine residue on its N-terminal end (numbered −1 and 0, respectively). Amino acids −1, 0, and 1, along with Nϵ2 of His-7, were found to coordinate a total of 2 Ni^2+^ ions (first and third). Another two Ni^2+^ ions (second and fourth) were coordinated by His-190 in the sifalimumab light chain. The fifth and sixth Ni^2+^ ions did not have histidine residues in the coordination sphere. Ni^2+^ ions were the only divalent metal ions present in the crystallization mixture, and could be identified by their fit to the corresponding electron density.

##### Analysis of Sifalimumab Binding to IFN-α2A

The interaction of immobilized IFN-α2A with sifalimumab was monitored using a KinExA 3000 instrument (Sapidyne Instruments, Boise, ID). IFN-α2A was first coated onto UltraLink Biosupport beads (Pierce, Rockford, IL) at concentrations of 5 and 10 μg/ml in 0.05 m NaHCO_3_, pH 9.0, overnight at 4 °C according to the manufacturer's instructions. Coated beads were then separated from unreacted IFN-α2A using a gentle pulse spin and blocked for ∼15 min at 22 °C with 1 m Tris, pH 8.0, bovine serum albumin, 10 mg/ml. The slurry was then spun and the blocking solution removed. The blocking step was repeated for 2 h at 22 °C. Beads were then resuspended in 27 ml of run buffer (phosphate-buffered saline (PBS), pH 7.4, 0.02% NaN_3_) and packed into a column. Typically, sifalimumab was prepared at concentrations of 40 and 200 pm. IFN-α2A was then titrated across these IgG solutions at concentrations of 313 fm to 16 nm and 980 fm to 50 nm, respectively, and incubated for 1–4 days at room temperature. The amount of free IgG in the samples was derived from the fluorescence signal obtained after the passing of Cy5-labeled goat anti-human IgG F(ab′)_2_ (typically 1 μg/ml; Jackson ImmunoResearch Laboratories, West Grove, PA) through the column. The dissociation constant (*K_D_*) was determined by fitting the individual equilibrium titration data to a 1:1 binding model using the KinExA Pro 1.0.3. software.

##### Receptor-ligand Competition by Sifalimumab

The ability of sifalimumab to inhibit the human IFNAR1/IFN-α2A and IFNAR2/IFN-α2A interactions was monitored using a ProteOn XPR36 instrument (Bio-Rad). The extracellular domain of IFNAR1 (MedImmune) was immobilized to the EDAC/Sulfo-NHS-activated surface of on a GLC biosensor chip (Bio-Rad) using standard amine coupling (200 nm in 10 mm sodium acetate buffer, pH 5.0) at a density of ∼4,800 resonance units according to the manufacturer's instructions. The extracellular domain of IFNAR2 (MedImmune) was also immobilized using standard amine coupling (50 nm in 10 mm sodium acetate buffer, pH 4.0) at a density of ∼2,000 resonance units. IFN-α2A and sifalimumab were prepared in PBS, pH 7.4, containing 0.005% Tween 20. Sifalimumab competition was assessed by 2 consecutive injections of IFN-α2A and a mixture of IFN-α2A and sifalimumab over the IFNAR1 or IFNAR2 surfaces. For IFNAR1, IFN-α2A was first injected at 200 μg/ml (30 μl/min for 120 s), which was followed by a second injection (30 μl/min for 120 s) of the IFN-α2A (200 μg/ml)/sifalimumab (100 μg/ml) mixture. For IFNAR2, IFN-α2A was first injected at 10 μg/ml (30 μl/min for 120 s), which was followed by a second injection (30 μl/min for 120 s) of the IFN-α2A (10 μg/ml)/sifalimumab (100 μg/ml) mixture. The extent of competition was derived from the additional binding detected from the second injection. All sensorgram data were processed by ProteOn Manager 3.1 software (Bio-Rad), and the binding graphs were prepared with Prism (GraphPad).

## Results and Discussion

### 

#### 

##### Sifalimumab Fab/IFN-α2A Three-dimensional Structure

We successfully determined the x-ray crystal structure of the complex between the Fab of an anti-human IFN-α therapeutic antibody (sifalimumab) and IFN-α2A. The corresponding refinement statistics are given in Table 1. Two sifalimumab Fab fragments in the asymmetric unit superimposed with an r.m.s. deviation of 0.31 Å (maximum displacement of 1.3 Å was for Cα/136 in the heavy chain and 1.2 Å for Cα/204 in the light chain). This value is well within the estimated overall coordinate error value of 0.34 Å. In addition, the elbow angles were calculated for both molecules as described (29) and separately estimated at 170.9° and 172.5°. These values are again well within the significance limit of 2–3° (29). Thus, we concluded that the 2 sifalimumab Fab molecules in the asymmetric unit were essentially identical. Both antigen molecules (IFN-α2A) exhibited a r.m.s. deviation of 0.6 Å when superimposed. However, the greatest differences occurred away from the Fab/IFN-α2A interface, and close to the IFN-α2A C-terminal region (maximum displacement of 3 Å was for Cα/156). As with sifalimumab Fab, both antigen molecules in the asymmetric unit could be considered essentially identical. Therefore, all subsequent descriptions of the antibody/antigen interface were made using 1 of the 2 complexes (namely chains A, B, and C in our PDB ID 4YPG).

**TABLE 1:** **Sifalimumab Fab/IFN-α2A model refinement statistics**

Statistics	
Resolution limits (Å)	20.0-3.0
*R* factor (free r factor)	0.206 (0.272)
R.m.s. deviation bonds (Å)	0.011
R.m.s. deviation angles (Å)	1.38
Residues in most favored region of {ϕ,ψ} space*^a^* (%)	89.5
Residues in additionally allowed region of {ϕ,ψ} space (%)	10.0
Residues in generously allowed region of {ϕ,ψ} space (%)	0.5
Number of protein atoms	9166
Number of non-protein atoms	53
Mean *B* factor (Model/Wilson), Å^2^	67/70

*^a^* The Ramachandran plot was produced using PROCHECK (45).

**FIGURE 1.**
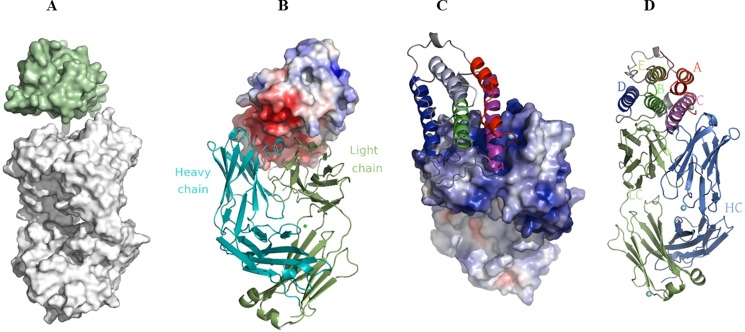
*A,* representation of the overall shape complementarity between the interacting surfaces of sifalimumab Fab and IFN-α2A. For clarity purposes, the antigen (*green*) and antibody (*white*) were shifted away from each other by 10 Å along the vertical axis. *B* and *C,* representations of the sifalimumab Fab·IFN-α2A complex showing charge complementarity between the 2 partners. The acidic surface of the antigen (shown in *red* surface in *panel B*) was found to exhibit very good complementarity to the basic surfaces of both variable regions of the antibody (shown in *blue* surface in *panel C*). The positive and negative electrostatic potentials were indicated in *blue* and *red*, respectively, and were calculated using APBS (Adaptive Poisson-Boltzmann Solver) plug-in in PyMOL. *D,* representation of the sifalimumab Fab (*bottom*)·IFN-α2A (*top*) complex. The IFN-α2A side of the contact interface mostly comprises parts of the B, C, and D helices of the antigen. *HC*, sifalimumab Fab heavy chain. *LC,* sifalimumab Fab light chain. This and subsequent illustrations were prepared using PyMOL.

The interface contributed by the sifalimumab Fab portion could best be described as a canyon, whereas IFN-α2A exhibited a remarkable shape complementarity to this groove as indicated in Fig. 1*A*. A shape complementarity of ∼0.674 between sifalimumab Fab and IFN-α2A was calculated using the “sc” program from the CCP4 suite (Collaborative Computational Project Number 4) (20). This represents a very high degree of complementarity between both partners. For comparison purposes, the shape complementarity between sifalimumab Fab heavy and light chains was estimated at 0.669. The charge complementarity between sifalimumab Fab and IFN-α2A is provided in Fig. 1, *B* and *C*. The basic surfaces of both variable regions of the antibody exhibited very good complementarity to the acidic surface of the antigen. Finally, we also noted that the side of the antigen of the sifalimumab Fab/IFN-α2A contact interface was formed mainly by parts of B (residues 49–69), C (residues 78–101), and D (residues 112–133) helices as illustrated in Fig. 1*D*.

**FIGURE 2.**
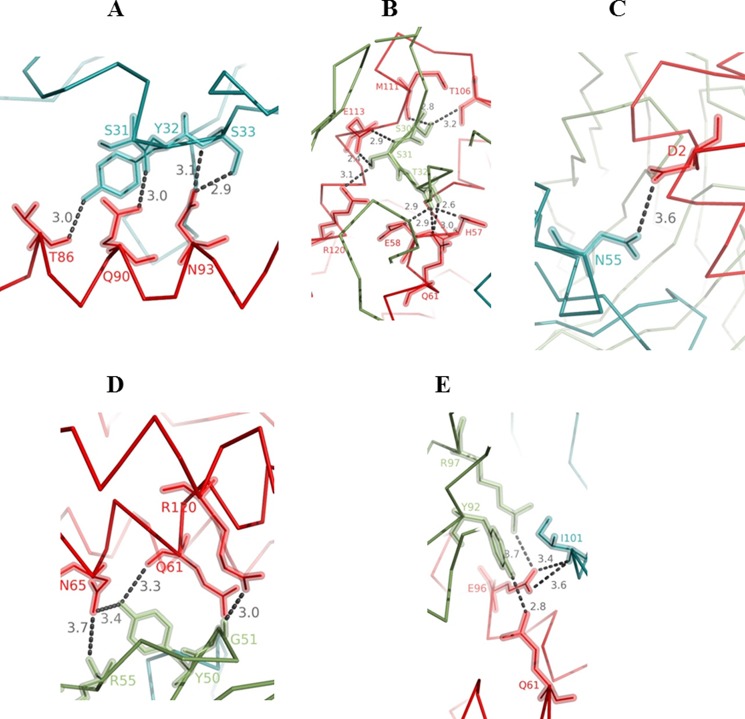
**Representations of the intermolecular contacts between IFN-α2A and sifalimumab around heavy chain CDR1 (*A*), light chain CDR1 (*B*), heavy chain CDR2 (*C*), light chain CDR2 (*D*), and heavy (*blue*) and light (*green*) chains CDR3 (*E*).** In all panels, the antigen is shown in *red*. Sifalimumab residues were numbered consecutively. *Dotted lines* represent hydrogen bonds.

The buried surface area upon formation of the complex between sifalimumab Fab and IFN-α2A was estimated at more than 1,300 Å^2^ (as calculated from the PISA server). The contact interface included ∼20 amino acids from each of the antibody polypeptides and ∼40 amino acids from the antigen. In particular, the heavy and light chains of sifalimumab Fab covered ∼600 and 700 Å^2^ of solvent-accessible area upon complex formation, respectively. The list of all hydrogen bonds between both chains of sifalimumab Fab and IFN-α2A can be found in Table 2. It is worth noting that the heavy and light chain CDRs of sifalimumab Fab contributed unequally to the interaction with IFN-α2A. Indeed, 4 intermolecular hydrogen bonds involved the heavy chain first CDR (CDRH1, Fig. 2*A* and Table 2), whereas 9 intermolecular hydrogen bonds involved the light chain first CDR (CDRL1; Fig. 2*B* and Table 2). Asn-55/Nδ2 in the second CDR of the sifalimumab Fab heavy chain (CDRH2) made a lone hydrogen bond with IFN-α2A Asp-2/Oδ2 (Fig. 2*C* and Table 2). The contribution of the second CDR of the sifalimumab Fab light chain (CDRL2) for binding to IFN-α2A appeared somewhat less specific, in that all corresponding intermolecular hydrogen bonds only involved the main chain CDRL2 atoms of Gly-51 and Arg-55 (Fig. 2*D* and Table 2). Finally, the spatially close CDRH3 and CDRL3 of the sifalimumab Fab created 2 intermolecular hydrogen bonds each with the antigen (Fig. 2*E* and Table 2). In CDRL3, Tyr-92/Oη and Arg-97/Nη2 created one hydrogen bond each with IFN-α2A Gln-61/Nϵ2 and Glu-96/Oϵ1, respectively. CDRH3 was only involved in contacts with IFN-α2A Glu-96/Oϵ1,2 through its main chain Ile-101/N atom. Interestingly, 2 intermolecular hydrogen bonds were mediated by one residue in the second framework of the sifalimumab light chain (Tyr-50). In summary, sifalimumab CDRL1 and CDRH1 made the largest contribution to formation of the high affinity complex between IFN-α2A and sifalimumab (*K_D_* shown in Table 3). Importantly, none of the side chains in sifalimumab CDRL2 and CDRH3 were involved in hydrogen bonds with IFN-α2A.

**TABLE 2:** **Summary of hydrogen bonds formed between sifalimumab Fab and IFN-α2A**

Sifalimumab	Distance (Å)	IFN-α2A
**CDRH1**		
Ser-31 (O)*^a,b^*	3.0	Gln-90 (Oϵ1)
Tyr-32 (Oη)	3.0	Thr-86 (O)*^b^*
Ser-33 (Oγ)	2.9	Asn-93 (Nδ2)
Ser-33 (N)*^b^*	3.1	Asn-93 (Nδ2)

**CDRL1**		
Ser-30 (Oγ)	3.2	Thr-106 (Oγ)
Ser-30 (Oγ)	2.8	Met-111 (O)*^b^*
Ser-31 (N)*^b^*	2.9	Glu-113 (Oϵ1)
Ser-31 (Oγ)	2.4	Glu-113 (Oϵ2)
Ser-31 (Oγ)	3.1	Arg-120 (Nη2)
Thr-32 (Oγ1)	3.0	Gln-61 (Nϵ2)
Thr-32 (O)*^b^*	2.6	His-57 (Nδ1)
Thr-32 (O)*^b^*	2.9	Glu-58 (Oϵ1)
Thr-32 (O)*^b^*	2.9	Gln-61 (Νϵ2)

**CDRH2**		
Asn-55 (Nδ2)	3.6	Asp-2 (Oδ2)

**FRL2***^c^*		
Tyr-50 (Oη)	3.3	Gln-61 (O)*^b^*
Tyr-50 (Oη)	3.4	Asn-65 (Nδ2)

**CDRL2**		
Gly-51 (O)*^b^*	3.0	Arg-120 (Nη2)
Arg-55 (O)*^b^*	3.7	Asn-65 (δ2)

**CDRH3**		
Ile-101 (N)*^b^*	3.4	Glu-96 (ϵ1)
Ile-101 (N)*^b^*	3.6	Glu-96 (Oϵ2)

**CDRL3**		
Tyr-92 (Oη)	2.8	Gln-61 (Nϵ2)
Arg-97 (Nη2)	3.7	Glu-96 (Oϵ1)

*^a^* Letters in parentheses refer to the corresponding interacting atoms.

*^b^* Main chain atoms.

*^c^* FR, framework.

**TABLE 3:** **Affinity measurement for the binding of sifalimumab to IFN-α2A** The dissociation constant (*K_D_*) was determined using a KinExa instrument as described under “Experimental Procedures.”

Molecule		95% confidence interval*^a^*
	*K_D_*	*pm*
Sifalimumab	44	27–65

*^a^* The 95% confidence interval indicated the range over which the measured *K_D_* is thought to vary due to the reproducibility of the instrument. The residual error between the fitted and theoretical curves was 2.7%.

Radhakrishnan *et al.* (12) observed that human IFN-α2B (99% identical to human IFN-α2A due to one amino acid difference, namely K23R) can form Zn^2+^ ion-mediated homodimers. Our model did not indicate such a dimerization mechanism because no ions were found to be shared between 2 contacting IFN-α2A molecules (despite the presence of Ni^2+^ in the crystallization mixture). This comforts the notion that the active form of IFN-α molecules is monomeric, as determined by Klaus *et al.* (13).

##### Implications for Sifalimumab Mechanism of Action

The type I IFN receptor (shared by all human type I IFNs) comprises 2 major transmembrane subunits, namely IFNAR1 and IFNAR2 (30, 31). IFN-α binds to IFNAR2 with a much faster *k*_on_ and slower *k*_off_ than those measured for IFNAR1 (32, 33). Therefore, a two-step assembling mechanism was proposed for formation of the tertiary IFN signal complex, in which IFN-α first binds IFNAR2 and then recruits IFNAR1 (32, 34). The present study provides important clues related to sifalimumab mechanism of action.

**FIGURE 3.**
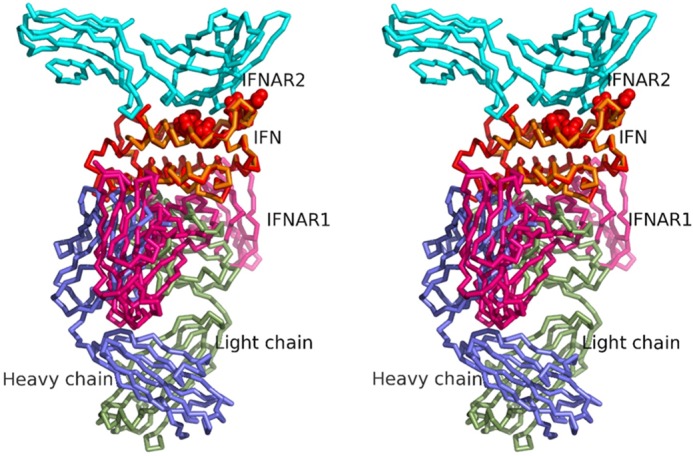
**Stereographic superimposition of the complex between IFN-α2A (*red*) and sifalimumab Fab heavy (*blue*) and light (*green*) chains with the x-ray-based model of the human IFN-α2·IFNARI·IFNAR2 ternary complex (*gold*, *pink*, and *cyan*, respectively; PDB ID 3SE3).** Human IFN-α2 residues Leu-30, Arg-33, Arg-144, Ala-145, Met-148, and Arg-149 identified as critical for binding to human IFNAR2 (37) are shown as *red spheres*. The superimposition was carried out through the Cα atoms of the IFN-α2 molecules using “lsqkab” (44).

**FIGURE 4.**
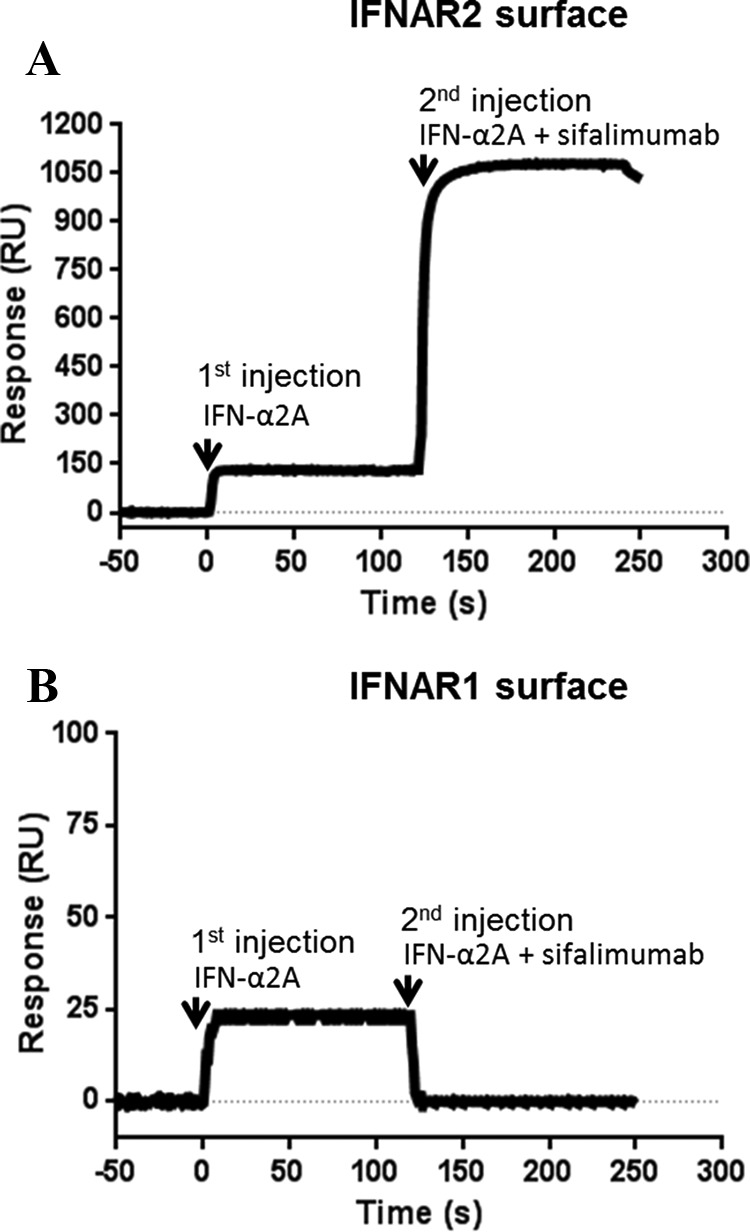
**Sifalimumab binding to human IFNAR2·IFN-α2A (*A*) and human IFNAR1·IFN-α2A (*B*) complexes.** Sifalimumab could bind to IFNAR2·IFN-α2A, but not IFNAR1·IFN-α2A complexes.

A number of studies have revealed critical residues on both IFNAR2 and IFN-α2 through mutagenesis, NMR, and x-ray crystallography (35–40). As can be observed when superimposing the x-ray structure of the human IFN-α2·IFNARI·IFNAR2 ternary complex (PDB ID 3SE3) (40) with the sifalimumab·IFN-α2A complex (Fig. 3), both sifalimumab and human IFNAR2 bind to opposite sides of the IFN molecule. In addition, various human IFN-α2 residues identified by Piehler *et al.* (37) as critical for binding to IFNAR2 (namely Leu-30, Arg-33, Arg-144, Ala-145, Met-148, and Arg-149) were also found to be on the opposite side of the sifalimumab binding site (Fig. 3). These data agree with the observation that sifalimumab can bind to IFN-α2A·IFNAR2 complexes (Fig. 4*A*), and, thus, rule out a mechanism of action in which the antibody interferes with the corresponding receptor/ligand interaction.

**FIGURE 5.**
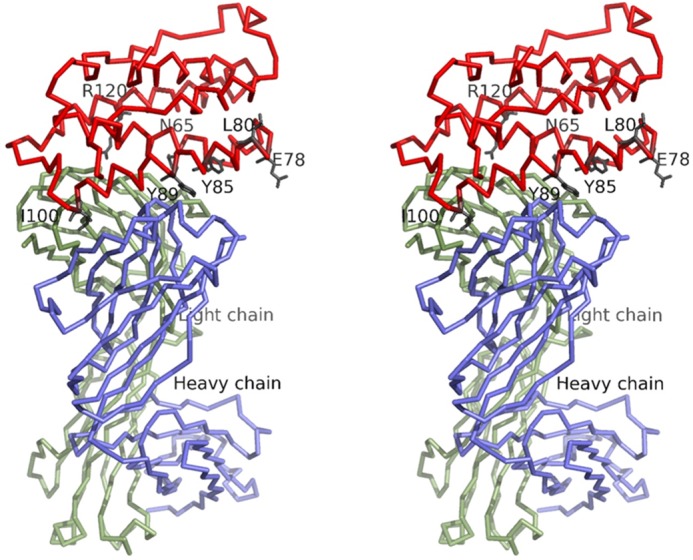
**Stereographic representation of the complex between IFN-α2A (*red*) and sifalimumab Fab heavy and light chains.** Human IFN-α2 residues Asn-65, Glu-E78, Leu-80, Tyr-85, Tyr-89, Ile-100, and Arg-120 identified as critical for binding to human IFNAR1 (33, 41, 42) are shown as *black sticks*.

Interestingly, binding of sifalimumab to IFN-α2A·IFNAR1 complexes could not be detected (Fig. 4*B*). In fact, sifalimumab actually inhibited the binding of IFN-α2A to immobilized IFNAR1, as observed from the drop in signal upon injection of the IFN-α2A/sifalimumab mixture. Indeed, the interaction of sifalimumab/IFN-α2A (*K_D_* of 44 pm) is much stronger than that of IFNARI/IFN-α2A (*K_D_* of 1.5 μm (33)). Therefore, sifalimumab interferon blocking activity appears to be caused by inhibition of IFN-α2 binding to IFNAR1. Our structure of the sifalimumab Fab·IFN-α2A complex provided a better understanding of this phenomenon. In particular, crucial amino acids for the IFN-α2/IFNAR1 interaction have been identified (33, 41–43), such as Asn-65, Glu-78, Leu-80, Tyr-85, Tyr-89, Ile-100, and Arg-120 in IFN-α2 B, C, and D helices. When mapped onto the structure of the sifalimumab·IFN-α2A complex, the region defined by these amino acids showed a significant overlap with the sifalimumab binding site (Fig. 5). Moreover, when the x-ray structure of the human IFN-α2·IFNARI·IFNAR2 ternary complex (PDB ID number 3SE3) (40) was superimposed with that of the sifalimumab·IFN-α2A complex (Fig. 3), it clearly appears that a large overlap exists between sifalimumab Fab/IFN-α2A and IFN-α2/IFNAR1 contacting surfaces. Collectively, these data suggest that sifalimumab precludes the IFN-α2/IFNAR1 interaction through *direct* steric hindrance.

In summary, we conclude that sifalimumab acts as a direct competitive inhibitor of the IFN-α2/IFNAR1 interaction. A coherent model of the mechanism of action for sifalimumab thus emerges, and indicates the antibody does not interfere with the first step in the response to IFN-α2, namely the ligand·IFNAR2 complex formation. Rather, we suggest that sifalimumab sterically interferes with the recruitment of IFNAR1 and prevents formation of the IFN-α2·IFNAR1·IFNAR2 ternary complex.

## Footnotes

3 The abbreviations used are:

CDR: complementarity-determining regions
PDB: Protein Data Bank
r.m.s.: root mean square
LC: light chain
HC: heavy chain.
